# Ultrasound-assisted extraction, optimization, and purification of total flavonoids from Daphnegenkwa and analysis of their antioxidant, anti-inflammatory, and analgesic activities

**DOI:** 10.1016/j.ultsonch.2024.107079

**Published:** 2024-09-23

**Authors:** Yi Xiang, Zheng Liu, Yanzhi Liu, Bin Dong, Changqing Yang, Hanhan Li

**Affiliations:** a School of Basic Medicine and Clinical Pharmacy, China Pharmaceutical University, 639 Longmian Avenue, Nanjing 21198, Jiangsu Province, PR China; b Department of Pharmacy, Foshan Women and Children Hospital, Foshan 528000, Guangdong Province, PR China; c School of Engineering, China Pharmaceutical University, 639 Longmian Avenue, Nanjing 21198, Jiangsu Province, PR China

**Keywords:** *Daphne genkwa*, Flavonoids, Ultrasonic, Response surface methodology, Artificial neural network

## Abstract

*Daphne genkwa (D. genkwa)* is the dried flower buds of a Chinese medicinal plant with multiple biological activities. Response surface methodology (RSM) combined with artificial neural network (ANN) techniques were utilized to optimize ultrasound-assisted extraction conditions for *D. genkwa*. Antioxidant activity and anti-inflammatory and analgesic properties of total flavonoids from *D. genkwa* (TFDG) were assessed. Optimal conditions involving ultrasonic power of 225 W, 30 min extraction time, 30 mL/g liquid–solid ratio, 60 °C extraction temperature, and 70% ethanol concentration yielded a maximum total flavonoids content (TFC) of 5.41 mg/g. After microporous resin purification, four specific flavonoids in *D. genkwa* were identified and quantified using high-performance liquid chromatography (HPLC). The TFDG demonstrated potent antioxidant activity, with a 94% rate of scavenging the 2, 2-diphenyl-1-picrylhydrazyl (DPPH). Furthermore, TFDG exhibited pain-alleviating properties in hot plate and acetic acid-induced writhing tests and noteworthy inhibitory effects on xylene-induced ear swelling in mice. The total flavonoids extracted by ultrasound had excellent biological activity. This establishes a foundation for further investigation into the potential medical value of *D. genkwa*.

## Highlights

• Optimize the ultrasonic-assisted extraction conditions of Daphne genkwa and determine five optimal extraction parameters.
• Identify and quantify four representative and biologically active flavonoid components in Daphne genkwa.
• The ultrasound extracted total flavonoids from Daphne genkwa have antioxidant activity and anti-inflammatory and analgesic properties.
• Provide a theoretical basis and experimental support for further developing the industrial application and medicinal value of Daphne genkwa.

## Graphical abstract

**Figure:**
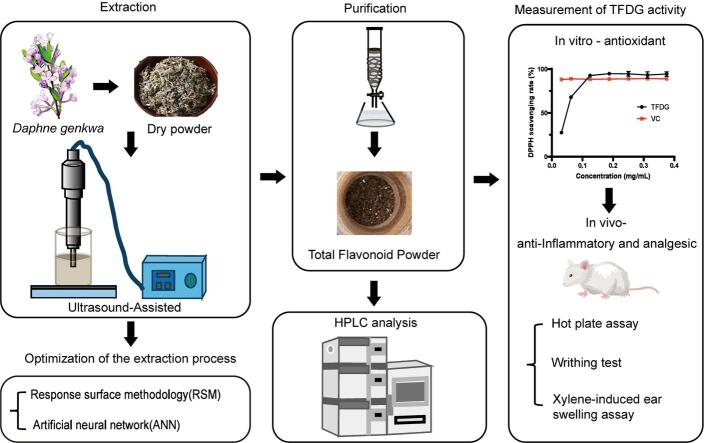

## 1 Introduction

*Daphne genkwa* Sieb. et Zucc. (Thymelaeaceae), the dried flower bud of *Daphne* species from the *Daphne* family, has a long history in China[1]. It is widely utilized in many formulae and possesses diuretic, insecticidal, and sore-treatment characteristics[2]. According to the reports available[3], [4], flavonoids, lignans, coumarin, and diterpenoids are the primary chemical components of *D. genkwa*. Among them, the main active components are flavonoids, including luteolin, apigenin, hydroxygenkwanin, and genkwanin[4], [5]. Current domestic and international pharmacological research has demonstrated the flavonoid components’ strong anti-inflammatory, antioxidant, analgesic, and anticonvulsant properties[6], [7], [8], [9], [10]. Specifically, research[8] has revealed that the alcohol extract of *D. genkwa* exhibits noteworthy analgesic effects, with the intensity of this effect being linked to the dosage of the extract. Sun etal.[6] further corroborated that the total flavonoids extracted from *D. genkwa* possess anti-inflammatory and analgesic effects that can combat rheumatoid arthritis.

Extraction is a crucial process for the utilization of natural plants. Bioactive components can be extracted effectively without sacrificing their functional quality through an ideal extraction technique[11]. Ultrasound-assisted extraction (UAE) is an advanced technique that preserves natural plant components’ structural and molecular properties during extraction[12]. It creates cavitation bubbles using ultrasonic waves, which collide and produce microjets with higher pressure, resulting in faster and more efficient extraction. UAE is a sustainable extraction method owing to its efficiency, low solvent usage, and high yield [13]. Additionally, numerous studies have shown that this method has promise for producing innovative functional goods or extracting highly potent bioactive chemicals from Chinese herbal medicine[14], [15], [16]. Several factors influence the UAE extraction process to varying degrees[12]. Thus, when optimizing the UAE extraction, the proper solvents, temperature, time, liquid–solid ratio, and sonication parameters (power and frequency) should be considered. Drawing upon mathematical and statistical tools, the optimized process is a beneficial methodology for experimental design, offering notable advantages in efficiency, conservation of time, resources, and effort. Response Surface Methodology (RSM) is a common statistical technique for optimizing intricate algorithms. It provides an effective and time-saving mathematical model that predicts several parameters and their interactions using quantitative data from appropriate experimental designs[17]. Artificial Neural Network (ANN) is an advanced neural system that manages inaccuracies and uncertainties while simultaneously considering multiple factors and conditions[18].

Despite the increasing interest in the potential bioactive effects of *D. genkwa*, further research is required to focus on optimizing extraction parameters and identifying bioactive constituents in *D. genkwa*. Therefore, this study employed a combination of RSM and ANN techniques to predict and optimize critical parameters, including ethanol concentration, extraction time, temperature, ultrasonic power, and liquid–solid ratio, to improve the efficiency of the ultrasound-assisted extraction process. This study aims to maximize the efficiency of total flavonoids extracted from *D. genkwa* while retaining their biological activity. Following optimizing the ultrasonic method for obtaining total flavonoids from *D. genkwa*(TFDG), the extract’s analgesic, antioxidant, and anti-inflammatory qualities were investigated. This research sought to contribute valuable insights into the pharmacological properties of this botanical species.

## 2 Materials and methods

### 2.1 Materials and chemicals

*D. genkwa* were collected from the planting base in Bozhou City, Anhui Province, China. Rutin standards (>98%) were acquired from Aladdin Reagent Co., Ltd. (Shanghai, China). Luteolin, apigenin, hydroxygenkwanin, and genkwanin standards (>99%) were purchased from Shanghai Yuanye Bio-Technology Co., Ltd. (Shanghai, China). Aluminum chloride was purchased from Sinopharm Chemical Reagent Co., Ltd. (Shanghai, China). Sodium nitrite and sodium hydroxide were purchased from Xilong Science Co. Ltd. (Shenzhen, China). Shandong Donghong Chemical Co., Ltd. (Shandong, China) provided the AB-8 macroporous resin. All other chemicals and solvents available were of analytical grade.

### 2.2 Ultrasound-assisted extraction

Referring to previous literature[19]for extracting total flavonoids (TF). After drying at 60 °C, the samples were ground, passed through a 100-mesh sieve, and kept at 4 °C. Weigh 1.0 g of the sample in a 50 mL conical flask, then add 30 mL of 70% ethanol. The bottle was immersed in an ultrasonic bath (PZ-1500T, Fangxu Technology Ltd., Shanghai, China) as the ultrasound source for this experiment. UAE was performed on an ultrasonic processor with an ultrasound power range of 15–1500 W. After extraction, the samples were cooled and weighed, and any weight loss was adjusted. Subsequently, the filtrate was extracted under reduced pressure.

### 2.3 Determination of the total flavonoids

By employing ${\mathrm{NaNO}}_{2}-\mathrm{Al}{\left({\mathrm{NO}}_{3}\right)}_{3}-\mathrm{NaOH}$ colorimetry, a method modified from a previous study[20], total flavonoid content (TFC) was determined. 0.5 mL of sample extract solution was mixed with 0.3 mL of 5% (m/v) sodium nitrite, 0.3 mL of 10% (m/v) aluminum nitrate, and 4.0 mL of 4% (m/v) sodium hydroxide solution. The mixed solution was incubated for 10 min. Then, the absorbance was measured by ultraviolet spectrophotometry (UV-7502C, Shanghai Xinmao Instrument Co., Ltd) at 510 nm. Rutin solution with different concentrations (0, 5, 10, 15, 25, and 30μg/mL) was used to calculate the standard curve. The total flavonoid concentration was calculated using an equation from the standard curve, and the results were expressed as mg rutin equivalents per g dry weight (mg RE/g DW) of *D. genkwa*.

### 2.4 Single-factor experiments

All other parameters remained unchanged while one factor was examined. The following were the observed factors and their levels: the liquid–solid ratio (10, 20, 30, 40, and 50 mL/g), the ethanol concentration (40%, 50%, 60%, 70%, 80%, and 90%), the extraction time (20mins, 30mins, 40mins, 50mins, and 60mins), the temperature (40 °C, 50 °C, 60 °C, 70 °C, and 80 °C), and the ultrasonic power (150 W, 175 W, 200 W, 225 W, and 250 W).

### 2.5 Response surface experimental design and statistical analysis

RSM was applied to evaluate the impact of independent variables, including ultrasonic power(A), extraction time (B), and liquid–solid ratio (C), on the total flavonoid content (mg/g). Using Design-Expert 13.0 software (Minneapolis, USA), the Box-Behnken design (BBD) method was used to design the experimental data. A three-level and three-independent variable design was done following the determination of the preliminary scope of extraction using single-factor tests. Table 1 lists the independent variables’ ranges and levels. The results of the single-factor experiment determined the independent variables and their ranges. The function response value (Y) was the total flavonoid content(mg/g).

**Table 1:** Box-Behnken design factor level, A: Ultrasonic power; B: Extraction time; C: Liquid–solid ratio

	Factor		
Level	A(/W)	B(/min)	C(/mL/g)
-1	175	40	10
0	200	50	20
1	225	60	30

### 2.6 Artificial Neural Network modeling

**Fig. 1:**
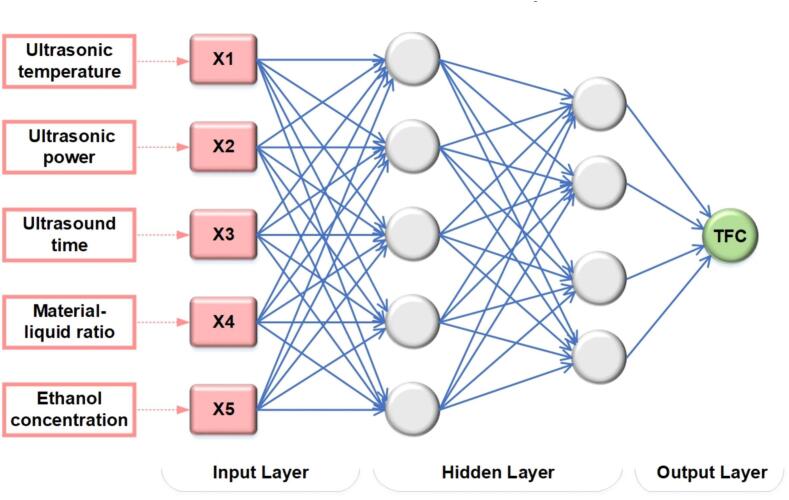
Optimal architecture of a developed ANN model.

The factorized nonlinear mapping between five input variables (X1, X2, X3, X4, and X5) and one output response (Y) was fitted using an artificial neural network (ANN). Y represents TFC, and the ultrasonic temperature, power, time, liquid–solid ratio, and ethanol concentration are represented by the variables X1, X2, X3, X4, and X5. An artificial neural network model was created to forecast the dependent variable Y by MATLAB’s $\mathrm{Neural}\;\mathrm{Network}\;{\mathrm{Toolbox}}^{\mathrm{TM}}$ [21]. As shown in Fig. 1, the model comprises a backpropagation feedforward (BPFF) model and a multi-layer perceptron (MLP). In ANN modeling, the data normalization preprocessing process is added to prevent the gradient from disappearing in the model training process caused by the significant difference in the numerical magnitude between dependent and independent variables. In forward propagation, independent variable weights and resulting output signals are used to activate each neuron, and the activation function is a sigmoid function. Simultaneously, the mean square error is utilized as an evaluation metric to estimate the number of hidden layers of the model to obtain the network structure with ideal performance. The error loss function between the expected and experimental outcomes is built during backpropagation.

### 2.7 Purification

AB-8 macroporous adsorption resin was used to purify total flavonoids[15]. After the pretreatment of macroporous resin, the column was loaded using the wet method. Elution conditions are as follows: sample concentration is 1.0 g/mL (crude drug concentration), 60% ethanol, and elution velocity is 2 BV/h after adsorption with 3–4 BV deionized water flushing after elution. The substance that was eluted with 60% ethanol was TFDG[22].

### 2.8 High Performance Liquid Chromatography

The chromatograph used was an High Performance Liquid Chromatography (HPLC) system (LC-2010A HT, Shimadzu Co., Kyoto, Japan) with a UV detector set to 338 nm. A C18 reverse-phase chromatography column (Galaksil®, 250 mm  × 4.6 mm × 5 μm) was utilized for precise quantitative determination of total flavonoid content in a 40 °C solvent system. Methanol (A) and an aqueous solution containing 0.5% acetic acid (B) made up the mobile phase. A gradient of 45% B (0–12 min), 45%-30% B (12–20 min), and 30%-45% B (20–25 min) was used to elute the column. 20μL was the injection volume and 1.0 mL·$\min^{-1}$ was the flow rate. A series of concentrations, consisting of four reference chemicals, were created by diluting the reference standard solution to build calibration curves. The identification of flavonoids in extracts from *D. genkwa* was based on comparing the retention time of standards and quantifying them using the external standard method.

### 2.9 In vitro antioxidant activity assay

The 2, 2-diphenyl-1-picrylhydrazyl (DPPH) radical scavenging assay was performed according to a slight modification of the previous method [23]. Samples at different concentrations (0.03, 0.06, 0.12, 0.19, 0.02, 0.03, and 0.38 mg/mL) were mixed with 0.2 mM DPPH methanol solution. After thorough mixing, incubate samples in the dark for 30 min. Absorbance was measured at a wavelength of 517 nm, with ascorbic acid (VC) as the reference standard. Three parallel experiments were conducted for each concentration, and the mean values were obtained. The DPPH scavenging rate (%) was determined according to the following formula:

(1) $$\%\mathrm{DPPH}\;\mathrm{scavenging}=\frac{A_{0}-(A_{s}-A_{b})}{A_{0}}\times 100$$

where $A_{0}$ is the absorbance of control (DPPH solution  + methanol); $A_{s}$ is the absorbance of the sample (DPPH solution  + sample solution); $A_{b}$ is the absorbance of the blank of the sample (sample solution + methanol).

### 2.10 Cell culture

The murine macrophage RAW264.7 cells, obtained from ${\mathrm{HyCyte}}^{\mathrm{TM}}$ (Suzhou, China), were cultured in a complete growth medium (DMEM with 10% fetal bovine serum). Incubate the dish in a cell culture incubator set at 37 °C, 5% ${\mathrm{CO}}_{2}$, and 95% relative humidity, and subculture it every three days.

### 2.11 Cell viability assay

RAW264.7 cells were adjusted to 2×$10^{5}$ cells/mL density and seeded at 2×$10^{4}$ cells per well in a 96-well plate. Following overnight incubation, different concentrations of TFDG (12.5, 25.0, 50.0, 100.0, and 200.0 mg/mL) were added to the wells. Following a 1-h incubation, LPS solution(1 μg/mL) was introduced to each well for overnight incubation (18 h). 10 μL of CCK-8(Sigma–Aldrich, USA) was added to each well and incubated for 2-4 h. Cell viability was calculated by quantifying the optical density at a wavelength of 450 nm. All experiments were conducted with six replicates (n  = 6).

### 2.12 Animals and Drug Administration

Kunming mice (17–23 g) were purchased from Qinglongshan Animal Co., Ltd. (Nanjing, China) and kept in a pathogen-free environment with unlimited access to food and water. Animals were kept at 20–25 °C and placed under a 12-h light/dark cycle. After acclimatization, mice were randomly assigned to groups: Control, TFDG (40.98 mg/kg), and Positive control (0.2 g/kg acetylsalicylic acid). The substances above were dissolved in 0.5% sodium carboxymethylcellulose (CMC-Na) and administered intragastrically at specified doses once daily for one week. Control groups received intragastric administration of 0.5% CMC-Na once daily for a week. China Pharmaceutical University’s Animal Care and Use Committee reviewed and approved all experimental protocols.

### 2.13 Anti-inflammatory and analgesic activity

Mice were subjected to a hot plate set at 55  ± 0.5 °C before grouping. Fifteen mice that responded within 30 s were selected and randomly divided into groups [24]. The latency to lick a hind paw or leap out of the apparatus was recorded for each group using a stopwatch. Pain thresholds of the mice in each group were determined after one hour of gavage administration on days 7 and 10, and the data were recorded.

Thirty mice were selected, as described by a previous study[25]. On the seventh day, each mouse was intraperitoneally injected with 0.1 mL of 0.8% glacial acetic acid after 1 h of gavage administration. The number of writhing reactions in mice was observed and recorded between 10 min and 20 min after injection.

The xylene-induced ear edema model was developed using the methodology previously described [26]. Thirty mice were administered for seven consecutive days. One hour after intragastric administration on the eighth day, the mice were anesthetized, and xylene was evenly applied to the front and back sides of the right ear, 0.02 mL per mouse. Four hours after inflammation, the mice were sacrificed, the left and right ears were cut off, and the round ear pieces were punched in the same part with a hole punch. The weight of the ear pieces was weighed, and the swelling inhibition rate was calculated.

### 2.14 Statistical analysis

The RSM experiment and statistical analysis were conducted using the Design Expert Software (Version 13, Minneapolis, MN, USA). The design and training of the models of the ANN were performed using the Neural Network ${\mathrm{Toolbox}}^{\mathrm{TM}}$ of MATLAB software (Ver. 8.6.0, Portola Valley, CA, USA). Data analysis was performed using GraphPad Prism 9.0 software, and significance analysis was performed using the one-way ANOVA and Dunnett test. P-values less than 0.05 (P<0.05) were considered statistically significant.

## 3 Results and discussion

### 3.1 Single-factor experiment

#### 3.1.1 Effect of extraction temperature

**Fig. 2:**
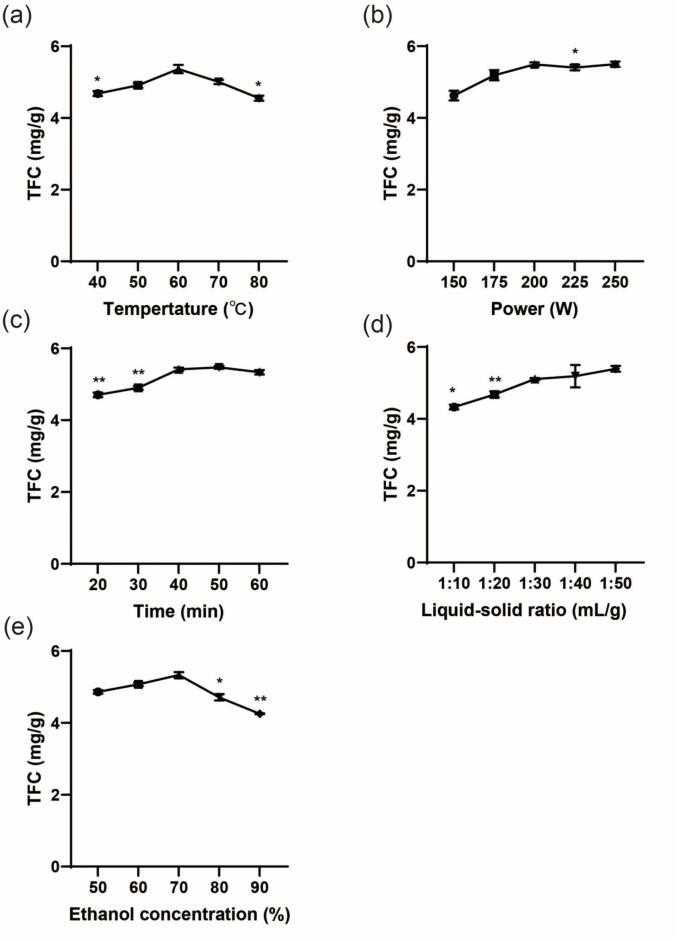
Effect of single factors on the total flavonoid content from D. genkwa. (a) temperature, (b) ultrasonic power, (c) extraction time, (d) liquid–solid ratio, (e) ethanol concentration (n  = 3). * P<0.05, **P<0.01 compared with the highest point in each group.

Fig. 2 illustrates the impacts of varying ultrasonic power, extraction time, ethanol concentration, liquid–solid ratio, and extraction temperatures on the total flavonoid content (TFC) from *D. genkwa*. The TFC gradually increased with rising temperature and peaked at 60 °C. The increase in temperature helps to accelerate the movement of bioflavonoids and solvent molecules through hydrogen bonding, thereby rapidly improving the exudation and diffusion process of flavonoids. This phenomenon speeds up the release and spread of flavonoids in plant materials[27]. However, above 60 °C, the extraction rate of total flavonoids tends to decrease. Excessively high temperatures can cause thermal decomposition of flavonoids[28]. Dehydration, dehydroxylation, and other chemical bond breakage may destroy the molecular structure of flavonoids[29], [30], [31]. Also, the polyphenolic structure of some flavonoids in *D. genkwa* may open the ring to generate a series of products with no biological activity. A suitable extraction temperature must be chosen to preserve the biological activity of the flavonoids in *D. genkwa* and maximize their extraction efficiency. So, 60 °C was selected for the further experiment.

#### 3.1.2 Effect of ultrasonic power

As the ultrasonic power increases, the TFC increases and begins to decrease when the ultrasonic power exceeds 200 W. With the rise of power, the cavitation effect is enhanced, and the degree of cellular fragmentation will intensify so that the extraction rate will substantially increase[32]. Within a certain range, the process of destroying cells and releasing flavonoids is facilitated by increased ultrasonic power in *D. genkwa*. Nevertheless, the propagation of shock waves is impeded by the formation of an increased number of bubbles when the ultrasonic power is excessive[33]. In addition, the bubbles may coalesce into more giant bubbles and undergo a weak implosion [34]. This is identical to what was reported by Fang etal.[35]. Besides, the high-intensity cavitation effect may lead to chemical bond breakage, molecular degradation, or free radical generation of some flavonoids in D. genkwa, all of which may destroy the structure of flavonoid compounds and reduce their activity. Moreover, as shown in Fig. 2(b), the results of one-way ANOVA analysis indicated no statistically significant difference in TFC between the ultrasonic power levels of 200 W and 250 W (P<0.05). To maximize the extraction rate while conserving resources, 200 W was considered to be the optimal ultrasonic power after a comprehensive evaluation.

#### 3.1.3 Effect of time

Time is in the process of ultrasonic-assisted extraction technology to extract the most critical parameters to consider. Different extraction times have different effects on the extraction efficiency of flavonoids, the stability of the compounds, the composition of the extracted components, and the extraction cost[36]. As ultrasonic extraction time increases, the cavitation effect is enhanced, leading to increased cellular fragmentation and substantially higher extraction rates[37]. Within a certain range, increasing the extraction time is conducive to the complete release and extraction of TF, and the extraction efficiency also increases. If the extraction time is too long, some poorly soluble flavonoids may also be extracted, but more impurities may be mixed in simultaneously. In addition, an extraction time that is too long may cause the flavonoids in *D. genkwa* to degrade in the solvent. Using a moderate extraction time, the cavitation effect of ultrasound is most effective, and the molecular structure of flavonoids remains stable. This conclusion aligns with the viewpoint of Wen etal. [16] and Li etal. [38]. The optimal time for ultrasonic extraction was 50 min.

#### 3.1.4 Effect of liquid–solid ratio

As the liquid–solid ratio grows, TFC exhibits a rise and, subsequently, a flat trend, culminating in a peak at 50 mL/g. When the liquid–solid ratio is small, the total flavonoids may not be able to be fully dissolved due to the insufficient extraction solution. Also, the solution can dissolve other substances, such as polysaccharides, decreasing the extraction rate[39]. This is analogous to the findings of LU etal.[40], suggesting that it could be attributed to increased solubility of non-flavonoids in the solution, competing with total flavonoids for solvent. A high liquid–solid ratio can improve extraction efficiency, but it increases solvent consumption and processing costs. One-way ANOVA showed that the liquid–solid ratio of 30 mL/g was not significantly different compared to 50 mL/g (P=0.19>0.05). Thus, the liquid–solid ratio of 30 mL/g was chosen to save cost and protect the environment while minimizing the utilization of organic solvents.

#### 3.1.5 Effect of Ethanol Concentration

With the rise in ethanol concentration, the total flavonoid content (TFC) generally shows an upward trend and reaches its peak at 70%. This trend may be attributed to ethanol’s good solubility and robust cellular permeability. Higher ethanol concentrations facilitate the dissolution of flavonoids in *D. genkwa* while reducing the proportion of polysaccharide impurities in the extract[41]. Meanwhile, when low-concentration ethanol is used as a solvent, it may cause hydrolysis or degradation of some flavonoids in *D. genkwa* due to the high water content. However, when the ethanol concentration exceeds 70%, the TFC gradually decreases. Very high ethanol concentrations are not beneficial for extraction due to the dehydration of plant cells, the shedding of collagen, and the denaturation of cell wall proteins[42]. Yu etal.[41] reported similar results and concluded that higher concentrations of ethanol prevent flavonoids from diffusing out of plant cells. So, moderate ethanol concentration can extract a variety of active flavonoids with different polarities from *D. genkwa*. At the same time, the extraction efficiency and solvent cost of various flavonoid compounds can also be considered. Hence, the optimal ethanol concentration for extracting flavonoids from *D. genkwa* is 70%.

### 3.2 Response Surface Experimental results

#### 3.2.1 Model building

For the BBD design, seventeen runs were performed to optimize the three parameters. Table 2 presents the factorial design-based TFC and experimental conditions. Under the specified testing conditions of an ultrasonic power of 225 W, extraction time of 40 min, and liquid–solid ratio of 30 mL/g, the maximum TFC reached 5.39 mg/g. The relationship between the response (TFC) and test variables (A, B, C) was determined by multivariate regression analysis using a second-order polynomial equation provided below:

(2) $$\begin{array}{cc} \mathrm{TFC} & =4.21-0.0097A-0.4507B+0.6982C\\ & -0.0941\mathrm{AB}+0.5315\mathrm{AC}+0.3055\mathrm{BC}\\ & -0.8453A^{2}+0.3149B^{2}+0.2483C^{2} \end{array}$$

**Table 2:** Box-Behnken experimental design and response values

Run	Factor			Response
No.	A: (W)	B: (min)	C: (mL/g)	TFC: (mg/g)
1	200	50	20	4.07
2	225	50	30	4.85
3	200	40	10	4.81
4	200	60	30	5.35
5	200	50	20	4.45
6	225	60	20	3.09
7	200	60	10	3.12
8	200	40	30	5.82
9	175	50	10	3.44
10	175	60	20	3.55
11	175	50	30	3.56
12	200	50	20	4.12
13	225	50	10	2.61
14	175	40	20	4.08
15	200	50	20	4.10
16	225	40	20	4.00
17	200	50	20	4.32

Table 3 lists the ANOVA results of the fitted models for all tested responses. Variance analysis revealed that the regression equation model had high significance(P<0.01). Furthermore, the selected model had an $R^{2}$ value of 0.9643, surpassing 0.9, confirming its reliability. The significant regression analysis showed that extraction time and liquid–solid ratio substantially affected TFC (P<0.01), while power did not significantly impact the TFC. The influence of each factor on TFC was ranked in descending order as C(liquid–solid ratio)> A(time) > B(ultrasonic power).

**Table 3:** ANOVA for response surface quadratic model.

Source	Sum of Squares	df	Mean Square	F-value	p-value	Significant
Model	10.59	9	1.18	21	0.0003	**
A	0.0008	1	0.0008	0.0135	0.9107	Not significant
B	1.63	1	1.63	29.01	0.001	*
C	3.9	1	3.9	69.63	<0.0001	**
AB	0.0355	1	0.0355	0.633	0.4524	Not significant
AC	1.13	1	1.13	20.17	0.0028	**
BC	0.3733	1	0.3733	6.66	0.0364	*
A^2^	3.01	1	3.01	53.71	0.0002	**
B^2^	0.4174	1	0.4174	7.45	0.0294	*
C^2^	0.2596	1	0.2596	4.63	0.0683	Not significant
Residual	0.3921	7	0.056			
Lack of Fit	0.2823	3	0.0941	3.43	0.1324	Not significant
Pure Error	0.1098	4	0.0274			
Cor Total	10.98	16				
R^2^	0.9643					

*:P<0.05; **:P<0.01.

#### 3.2.2 Interaction analysis

Combined with the residual distribution plot, the TFC of the factorial and central points was roughly in a straight line, indicating a good correlation between them. The experimental data, dispersed around the theoretical line, demonstrates that outcomes align with the model’s predictions. Additionally, the response surface and contour map are used to analyze and assess any two factors that affect TFC interaction. AC, BC interaction terms analysis of variance (P<0.05), indicating that the influence of each factor on the TFC was significant. There was no significant difference in the TFC in the AB interaction term (P>0.05). The lack of fit of the equation is not statistically significant (P>0.05), suggesting that the equation is a suitable fit and can be utilized to forecast the result of ultrasonic-assisted extraction for total flavonoids in *D. genkwa*.

**Fig. 3:**
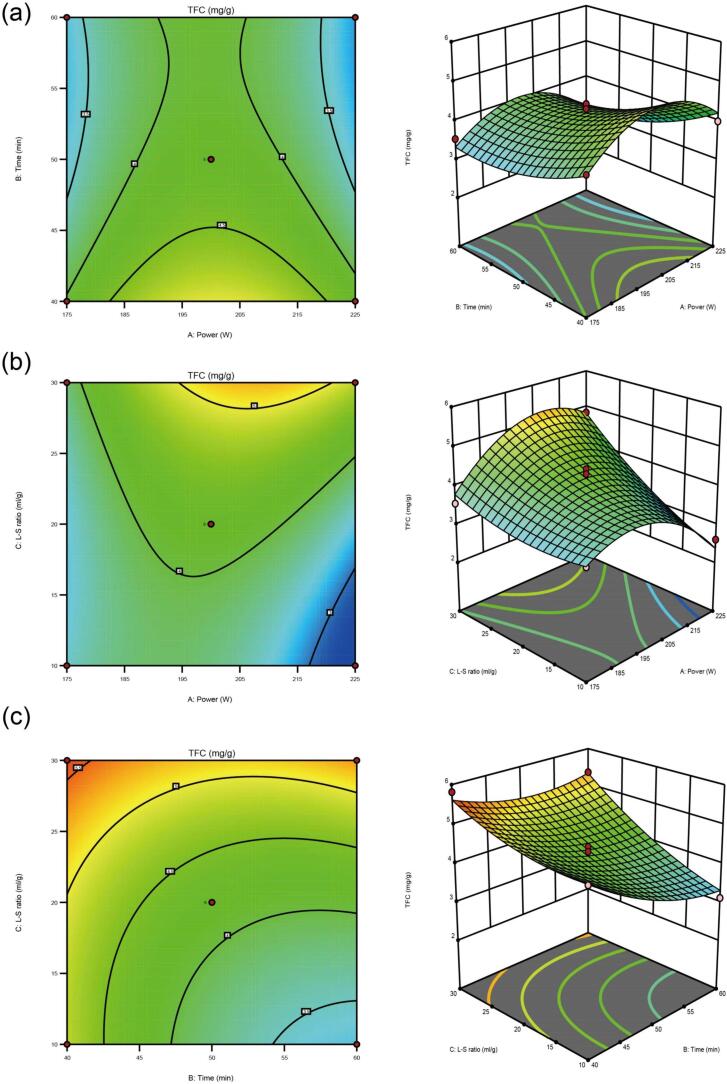
Response surface 3D plots and contour plots show the significant interactive effect of extraction variables on the total flavonoids of *D. genkwa*. (a) ultrasonic power and time, (b) liquid–solid ratio and ultrasonic power, (c) liquid–solid ratio and time.

Design-Expert software was implemented to generate three-dimensional response surfaces, visually representing the interactions between factors for the optimal response value. Fig. 3 illustrates the interaction between two independent variables and presents the perturbation plot of the TFC from *D. genkwa*. There was an interaction among extraction temperature, extraction time, and liquid–solid ratio. The impact of the interaction between power and liquid–solid ratio was the most significant. Then, there was the time and liquid–solid ratio, while the correlation between power and time was insignificant.

#### 3.2.3 Validation of the response surface experimental model

Based on the optimization test results, the optimal extraction process of flavonoids from *D. genkwa* includes the following: ultrasound power of 225 W, liquid–solid ratio of 30 mL/g, ethanol concentration of 70%, extraction time of 40 min, and extraction temperature of 60 °C. Three validation tests were conducted to verify that the best extraction parameters were applicable in these conditions. The experiment yielded an average value of 5.07  ± 0.24 mg/g (n = 3), with a relative error of 5.75% between predicted and actual values, indicating the efficiency and precision of the RSM and regression models. Thus, the RSM model proves effective in extracting the total flavonoids from *D. genkwa*.

### 3.3 Artificial neural network model

In the artificial neural network model, we first produced 42 experimental datasets, and 76% (32 groups) of the labelled data were used for network training and 24% (10 groups) for network testing by random sampling. At the same time, we evaluate the algorithm through the network performance, using the model mean square error value as the evaluation index. The optimal prediction performance was achieved when the hidden layers of the model were 9, and the mean square error was 0.03. The input layer is set up with five nodes, whereas the output layer is configured with one node.

In the training process, after 13 rounds of training, the loss error decreased and converged to 0.003, reaching the preset range. The output predicted values obtained by feeding the test set into the model are shown in Table 4. The model values of the artificial neural network were close to the experimental values.

**Table 4:** ANN models on TFC

Run	Parameter					TFC (mg/g)		
No.	X1: (°C)	X2: (W)	X3: (min)	X4: (mL/g)	X5: (%)	Experimental value	RSM Predicted	ANN Predicted
1	60	200	60	10	70	3.12	3.32	3.07
2	60	200	40	30	70	5.22	5.62	5.14
3	60	175	50	10	70	3.44	3.46	3.95
4	60	200	30	30	70	5.40	6.71	5.20
5	60	225	30	30	70	5.41	6.57	5.84
6	60	250	30	30	70	5.37	4.75	5.44
7	60	200	20	30	70	4.71	8.43	4.96
8	60	200	30	30	70	5.20	6.71	5.20
9	70	200	40	30	70	4.97	5.62	5.05
10	80	200	40	30	70	4.88	5.62	4.90

X1: extract temperatures; X2: ultrasonic power; X3: extraction time; X4: liquid–solid ratio; X5: ethanol concentration.

The predictive capability of RSM and ANN models was assessed and compared using different parameters, including RMSE (Root Mean Square Error) and MAPE (Mean Absolute Percentage Error)[43]. For better modeling, having lower RMSE and MAPE values is preferable. The Mean Squared Error (MSE) and RMSE numbers measure the model’s accuracy in terms of its absolute fit. The MAPE values stand for mean absolute percentage error. Table 5 demonstrates that the ANN model outperformed the RSM for having lower RSE, RMSE, and MAPE values. ANN outperforms RSM in terms of experimental response fitting, prediction, and mathematical modeling.

**Table 5:** Comparative error analysis of RSM and ANN models

Parameters	ANN	RSM
MSE	0.11	58.68
RMSE	0.33	7.66
MAPE(%)	5.01	20.34

### 3.4 High performance liquid chromatography analysis

**Fig. 4:**
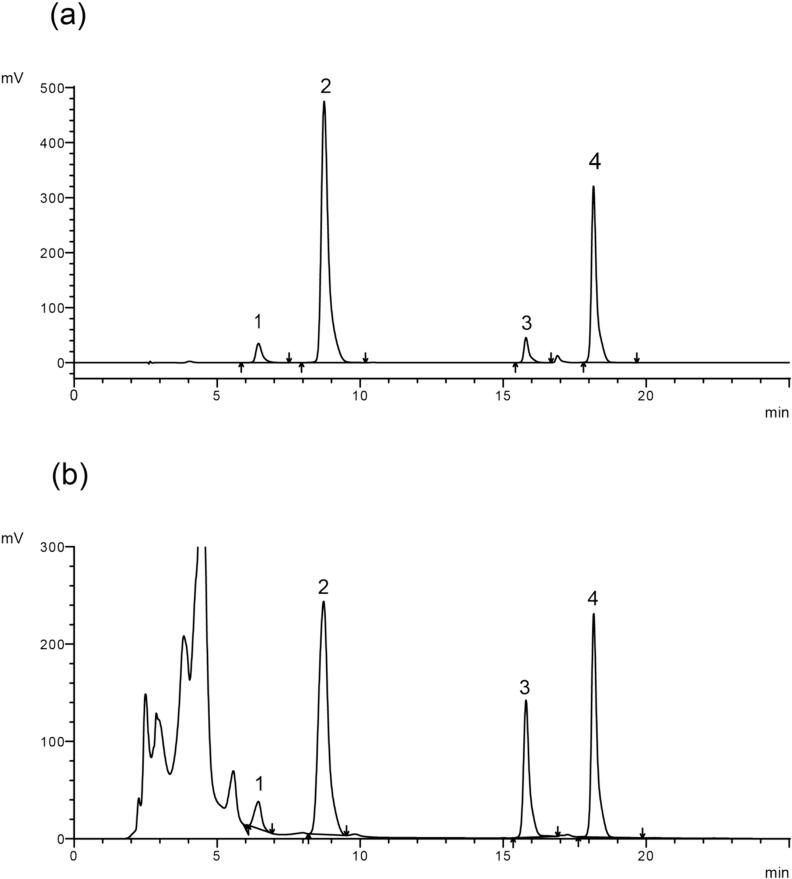
HPLC chromatogram of the main flavonoids in TFDG. (a) mixed standard; (b) test sample. The contents of four compounds are Compound1: luteolin; Compound2: apigenin; Compound3: hydroxygenkwanin; Compound4: genkwanin.

The specific four kinds of flavonoid ingredients were detected. The chromatogram is shown in Fig. 4. Four types of flavonoids, including luteolin, apigenin, hydroxygenkwanin, and genkwanin, were identified, and their elution times were 6.43, 8.72, 15.78, and 18.16 min, respectively. According to the HPLC quantitative analysis results, the total contents of the four flavonoids were 235.46 mg/g (plant material, dry weight). The purity of the four flavonoids was as follows: 11.91% overall, with luteolin at 1.48%, apigenin at 11.04%, hydroxygenkwanin at 4.82%, and genkwanin at 6.48%. The present experimental method increased the purity by 100.2% compared to our laboratory’s previous conventional extraction method. It proved that the optimized extraction process increased the total flavonoid content.

### 3.5 Effect of ultrasound on total flavonoids

Currently, the extraction methods for total flavonoids from *D. genkwa* are predominantly traditional, with many studies employing heating reflux extraction[44], [45], [46]. While this method is straightforward, it has drawbacks such as prolonged extraction time and high energy consumption compared to UAE. In this study, the UAE method increased the extraction rate from 5.89% to 6.74% compared to the heating reflux extraction method used in previous study[47]. It also saved a lot of operating time and reduced the use of more organic reagents. UAE leverages cavitation, thermal, and mechanical phenomena, making it a highly promising technology for extracting flavonoids[48], [49], [50]. The collapse of cavitation bubbles and propagation of sound waves induce complex effects such as plant cell matrix fragmentation, pore formation, and shearing[51]. These effects, acting individually or synergistically, enhance the dissolution of flavonoids. The cavitation-induced pores further facilitate the release of flavonoids from the cell matrix[52]. Consequently, UAE significantly improves the dissolution of intracellular compounds from *D. genkwa*, enabling efficient collection and purification of flavonoids. This study reached a similar conclusion, demonstrating that UAE improved the extraction efficiency of TFDG.

### 3.6 Antioxidant Activity of TFDG

**Fig. 5:**
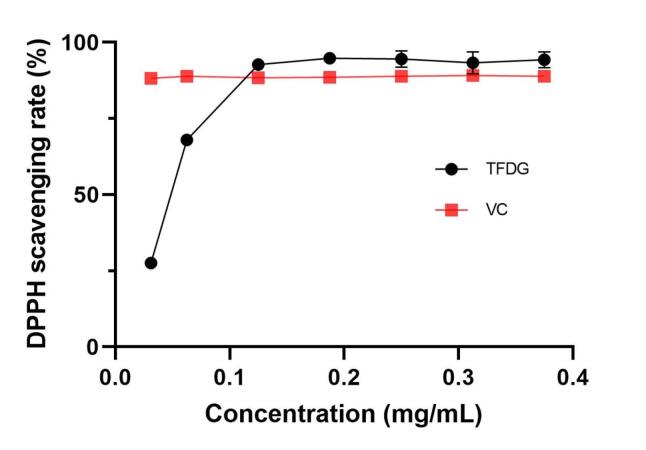
DPPH scavenging activities of TFDG (n = 3).

Antioxidant activity is an essential property of natural products[53], [54] and should be considered, especially when utilizing new environmentally friendly extraction methods. Recent research has focused on flavonoids’ in vitro antioxidant properties[55], [56]. As shown in Fig. 5, TFDG demonstrated a notable inhibitory impact on DPPH free radicals. This inhibitory effect became more pronounced as the concentration of TFDG increased, eventually reaching a level comparable to the inhibitory effect of vitamin C (VC). At a 0.125 mg/mL concentration, the TFDG scavenging rate reached 92.73%, demonstrating a strong scavenging capability. For the concentration exceeding 0.125 mg/mL, the scavenging rate of total flavonoids stabilized at about 94%. These results indicate that TFDG extracted using ultrasound-assisted extraction has strong antioxidant activity, highlighting the key advantages of ultrasound technology.

### 3.7 In vitro toxicity assay

**Fig. 6:**
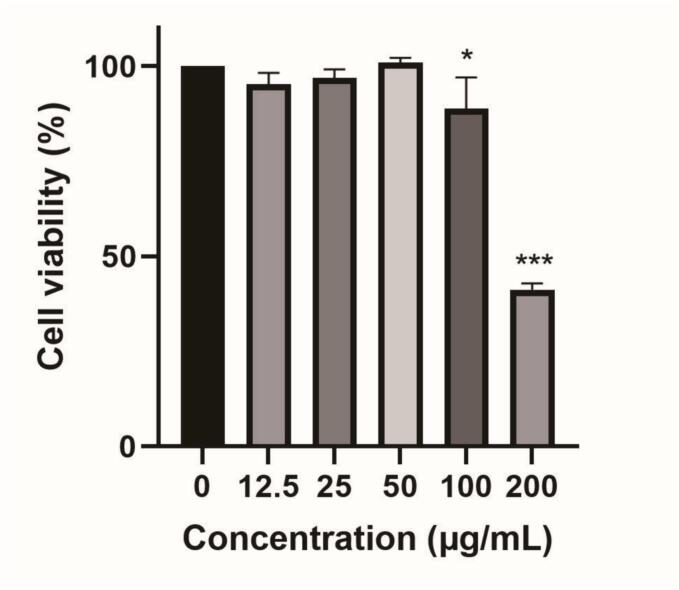
Cell viability of RAW264.7 cells exposed to TFDG (n  = 6). *P<0.05, ***P<0.001 compared with control group.

The non-toxic dose test of a medicinal product serves as the foundation for assessing its effectiveness and forms the basis for evaluating the impact of plant extracts. RAW264.7 cells, a versatile monocyte/macrophage cell line, have robust phagocytic capability, cytokine release, and other functions. These attributes make them highly valuable for studying inflammatory responses and drug screening. The safe concentration of TFDG on RAW264.7 cells was determined by CCK-8 cell viability assay, as presented in Fig. 6. TFDG treatment at 200 μg/mL significantly decreased cell viability in RAW264.7 cells compared to the control group (P<0.01). TFDG treatment of RAW264.7 cells at 100 μg/mL resulted in no significant difference in cell viability, indicating no toxicity at this dose. Thus, 100 μg/mL of TFDG was selected as the maximum concentration in vitro experiments.

### 3.8 Anti-inflammatory and analgesic assay

**Fig. 7:**
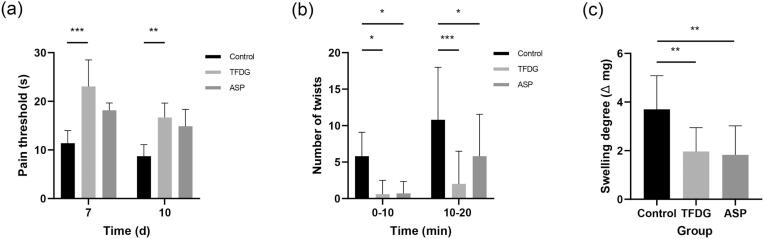
Antinociceptive and anti-inflammatory effect of TFDG. (a) the hot plate assay (n  = 5), (b) the writhing test (n  = 10), (c) the Xylene-induced ear swelling assay (n  = 10). *P<0.05, **P<0.01, ***P<0.001.

The hot plate method represents a foundational approach to evaluating the central analgesic effects of drugs, utilizing thermal stimulation to measure pain thresholds in mice[57]. As illustrated in Fig. 7(a), analysis of pain thresholds following the administration of TFDG revealed a substantial increase on the seventh day (P<0.01) and tenth day (P<0.01) post-treatment compared to the control group. Notably, no significant differences in pain thresholds between the seventh days post-administration relative to the positive control group were observed. These results highlight the efficacy of TFDG in enhancing pain thresholds in response to thermal stimuli, demonstrating superior analgesic effects compared to acetylsalicylic acid on both the seventh and tenth days. This study underscores the potent analgesic properties of TFDG in alleviating pain induced by thermal stimuli in mice, suggesting its potential as a promising candidate in pain management research.

The acetic acid writhing method is a well-established chemical stimulation technique utilized to assess the peripheral analgesic properties of test substances[58], [59], [60]. The nociceptive mediators released in this chemical nociception model excite nerve terminals and cause visceral pain[61]. In Fig. 7(b), it is demonstrated that TFDG notably decreased the number of twisting reactions following the intraperitoneal administration of acetic acid. This reduction in twisting was observed within 10 min and 20 min post-administration in mice, and the number was statistically significant when compared with the control group (P<0.05,P<0.01), respectively. These results indicate that TFDG exhibits a significant inhibitory effect on pain responses elicited by acetic acid stimulation, highlighting its potential as a peripheral analgesic agent in this experimental model.

Xylene, a chemical inflammatory agent, can induce acute inflammation by triggering the release of inflammatory mediators within tissues by enhancing local capillary permeability at the exposure site [62]. This process promotes the infiltration of inflammatory cells, leading to tissue edema[59]. Fig. 7(c) illustrates the inhibitory effect of TFDG on xylene-induced ear swelling in mice. TFDG significantly reduced xylene-induced ear swelling in mice compared to the control group. Notably, the positive drug acetylsalicylic acid reduced xylene-induced ear swelling by 50.12%, while TFDG exhibited a more significant inhibitory effect of 58.18%. These findings suggest that the inhibitory effect of TFDG is comparable to that of acetylsalicylic acid. Overall, the results demonstrate the promising anti-inflammatory potential of TFDG in this experimental setting.

UAE can markedly improve the extraction rate of biologically active substances, including polyphenols, flavonoids, and other natural compounds[63], [64]. Flavonoids are well-known for their ability to scavenge free radicals, inhibit inflammatory pathways, and modulate pain signaling mechan- isms[65], [66]. Hence, by enhancing the extraction of flavonoids from *D. genkwa*, ultrasonication can lead to extracts with more potent antioxidant and anti-inflammatory properties. Overall, the findings of this study indicate that the high content and purity of TFDG obtained through ultrasound-assisted extraction, achieved with shorter extraction times and lower energy requirements, largely preserve its antioxidant, anti-inflammatory, and analgesic effects.

## 4 Conclusion

*D. genkwa* is a Chinese medicinal plant that performs various biological activities. In this study, we successfully optimized the parameters for the ultrasound-assisted extraction of TFDG and demonstrated their significant antioxidant, anti-inflammatory, and analgesic activities. The optimal extraction conditions were determined to be 225 W ultrasonic power, 30 min extraction time, 30 mL/g liquid–solid ratio, 60 °C extraction temperature, and 70% ethanol concentration, yielding a maximum total flavonoid content of 5.41 mg/g. TFDG exhibited robust antioxidant effects in vitro and showed considerable anti-inflammatory and analgesic properties in vivo. This study presents an innovative and effective strategy for optimizing plant extraction processes by combining RSM with ANN. These findings enhance our understanding of the bioactive properties of *D. genkwa* and provide valuable insights into the potential therapeutic use of its flavonoid components. Further research is warranted to scale up this extraction process and explore additional pharmacological applications.

## Declaration of Competing Interest

The authors declare that they have no known competing financial interests or personal relationships that could have appeared to influence the work reported in this paper.
